# Data and Information Privacy as a Human Right: A Qualitative Study of its Perceived Impact on Mental Health

**DOI:** 10.17505/jpor.2026.29049

**Published:** 2026-03-26

**Authors:** Nikita Benjamin

**Affiliations:** Department of Psychology, Northumbria University, Newcastle-upon-Tyne, United Kingdom

**Keywords:** Data Privacy, Right to Privacy, Human Rights, Mental Health, Surveillance, Bullying, Thematic Analysis

## Abstract

The Right to Privacy has ancient and historical roots. Previous and ongoing research make it quite evident that privacy is an essential human need central to the development and integrity of the individual and society. The Universal Declaration of Human Rights identifies privacy as a basic human right that serves as a foundation for several other rights. With the all-pervasive acceptance of digital technology in the daily lives of people, the need to understand the impact of digital privacy is even more pertinent. This is especially true considering global ethical violations of data and no cohesive rules on privacy. Nine participants from varied professional backgrounds were interviewed in order to examine how they perceive the impact of Data and Information Privacy on their Mental Health. Thematic Analysis generated three superordinate themes: (1) Convenience; (2) Psychological and emotional impact; and (3) Distrust of state machinery and corporations. Each theme comprised subthemes. Results highlight that it is almost impossible to operate in the digital age without sharing one’s information online. Being aware of the risks involved, participants choose to balance both the negative impact and benefits of sharing information online. The significant negative effects experienced by the participants are reflective of ongoing incidents and research worldwide. The findings of this study indicate that the impact of Data Privacy is extremely important and requires continuing research as it impacts both the individual and society.

## Introduction

Privacy is described as having the ability to decide for ourselves the means, the timing and degree of information about us being shared with others (Westin, 1967). The concept of privacy has roots that go back to the time of Aristotle, where he distinguished between public and private aspects of one’s life (DeCew, 2002). The demarcation between the private and the public has been used to indicate the distinction between areas for governmental authority versus areas for self-regulation. This means privacy can refer to areas which can be considered inappropriate for governmental interference and also areas for solitude and limited access. (Roessler & DeCew, 2023).

According to a law review article published by Warren and Brandeis (1890), it was necessary for the legal system to recognize ‘the right to privacy’, as revealing private information about an individual to others could cause injury to the individual’s ‘estimate of himself’ (Glancy, 1979). However, there have been many critiques of privacy. Notably, Judith Jarvis Thomson (1975) pointed out that there was limited agreement on the subject and that ultimately the right to privacy was merely a cluster of rights wherein many cases, what may seem like a violation of privacy can be explained by an overlap of various other rights. Another critique, by Richard Posner (1981), argued that organizational and corporate privacy mattered more than personal privacy, in the interest of enhancing the economy.

Conversely, many theorists have emphasized the deep relationship between privacy and intimacy. According to Fried (1970), privacy is fundamental to personal development and enables people to form intimate friendships and relationships based on trust and respect. And these quality relationships are only possible if privacy is accorded to each other. Therefore, privacy is essential to the development of one’s personality and integrity as a person. Similarly, according to Gerstein (1978), it is necessary for intimacy to exist without any interference, if we are to comprehensively experience our lives spontaneously without feeling ashamed or observed. And for this to happen, privacy is a necessary condition in interpersonal relationships and communication. Rachels (1975) argues that privacy is essential for maintenance of diverse interpersonal social relationships and is not limited only to the intimate ones. According to his analysis, privacy allows us to maintain different types of behaviour with varied social relationships by enabling us to control access to information about ourselves in the way we want. Privacy is also essential for self-expression and social freedom (Schoeman, 1992). Amid all these varied views, there seems to be an absence of clear guidelines on the importance of privacy. However, there appears to be agreement that in a liberal democratic society the significance of privacy mostly lies in the protection of individual interests such as information, spaces, choices, freedom, and autonomy in the personal realm (Roessler & DeCew, 2023; Reiman, 2004; Roessler, 2005; Moore, 2010; Allen, 2011).

Alan Westin’s Privacy Theory (Westin, 1967), is considered seminal in the development of contemporary privacy laws. He defined privacy as “the claim of individuals, groups, or institutions to determine for themselves when, how, and to what extent information about them is communicated to others” (Westin, 1967). He describes four privacy functions namely

- *personal autonomy,* or the desire to not be exposed or manipulated by others and the ability to make life choices without external pressure whilst also testing varied ideas and opinions in social contexts of one’s own choosing,
- *emotional release* defined as an ability to take a break from role demands and the emotional stimulation of everyday life,
- *self-evaluation* denoting self-reflection while processing one’s experiences and developing one’s own identity, values and personal goals and lastly,
- *limited and protected communication* which refers to setting interpersonal boundaries and protecting the exchange of information with trusted peers and the freedom to choose who to be vulnerable with.

Westin also described four different states of privacy which facilitate the functions of privacy to be achieved. These are; *solitude* or not sharing information with others, *intimacy* or sharing information only with specific people, *anonymity* meaning information cannot be directly connected to a particular individual and lastly, *reserve* defined as limited information disclosure to others and barrier against unwanted intrusion (Marky, 2023).

Intimacy and privacy are deeply related, and privacy is essential for establishing and maintaining intimacy (Roessler & DeCew, 2023; Cohen, 2002; Gerstein, 1978; Fried, 1970; Gerety, 1977). Privacy is essential for us to maintain varied and diverse interpersonal social relationships and is not limited only to the intimate ones (Rachels, 1975). According to Altman (1975), privacy is a social process wherein an individual’s desired level of privacy within specific contexts might not always be the level achieved in reality. While too much privacy may lead to social isolation and feelings of loneliness, too little privacy may cause modification of behaviour. Too much contact and access to information can feel like an invasion of the self and consequently impact one’s sense of well-being (Pedersen, 1997). Despite these dilemmas, people may choose to disclose personal information when it enables them to maintain relationships and collaborate with others to meet common goals (Hsu et al., 2022.) The complex interplay between sharing of information with others, while being aware of the risks involved to one’s privacy, especially on digital platforms, is known as the privacy paradox (Barth et al., 2019).

In the age of technology, digital privacy forms an extremely important part of services and interactions on the internet and can be further divided into information privacy, communication privacy and individual privacy (Hung & Wong, 2009). According to Hung and Wong (2009), Information Privacy denotes the privacy that individuals should have in determining the ways in which their digital information can be collected and used. Communication Privacy refers to the freedom or right where digitally communicated information should only be accessible to the original intended recipient. And lastly, Individual Privacy refers to the idea that individuals should be able to choose what kind of information they want to be exposed to, on the internet.

Article 12 of The Universal Declaration of Human Rights identifies the right to privacy as a basic human right that serves as a foundation for several other rights (United Nations [UN], 1948). This was further endorsed in the International Covenant of Civil and Political Rights (UN, 1976). In December 2013, a resolution on the ‘right to privacy in the digital age’ was adopted by the United Nations, which reaffirmed that the same human rights standards should apply to surveillance, collection of personal information and interception of communication (UN, 2013). In a recent report published by the United Nations Human Rights Council (UNHRC), there was special focus on the right to digital privacy (UNHRC, 2022). Furthermore, the United Nation’s Secretary General’s (UNSG) report published in 2020, laid down a roadmap for the protection of digital privacy (UNSG, 2020).

Disclosing personal information and data on online platforms and social networking sites has become a regular occurrence for most of us, in both our personal and professional lives. Users remain willing to disclose information online because it gives them access to relevant and important information and leads to immediate problem solving conveniently (Hsu et al., 2022; Magedanz & Simões, 2009; Dienlin & Trepte, 2015). Furthermore, people may also disclose personal information on Social Networking Sites in order to collaborate with others to meet common goals, access knowledge and develop and maintain relationships with others (Hsu et al., 2022). Consequently, a massive amount of our personal information is shared and stored on the internet (Stevic et al., 2022). Most people lack adequate knowledge and control about who has access to their personal information online and how it is being used (Joinson & Paine, 2007). A perceived loss of control and protection of our personal information can be defined as privacy concerns (Deinlin, 2014). Multiple studies indicate that concerns about misuse of one’s personal information are usually about identity theft, financial fraud, advertising, cyber bullying, sexual harassment (Dienlin & Trepte, 2015; Mesch, 2009; Milne et al., 2009, Stevic et al., 2022). Many of these concerns are valid and have a basis. There have been multiple instances of data misuse, loss of privacy or surveillance without explicit consent. For example, in 2018, the Cambridge Analytica-Facebook scandal broke which revealed that the personal information of millions of Facebook users was harvested for psychographic profiling without their explicit consent and then used for targeted political advertising for the United States (US) Presidential Election (Cadwalladr, 2018; Lapowsky, 2019). In a massive data breach of unprecedented scale, the sensitive personal information of millions of US citizens was compromised when Equifax, suffered a major data security breach (Marcus, 2018). The information leaked included sensitive details like names, date of birth, credit card details, social security number, driver’s license numbers etc. (Marinos & Clements, 2018).

In many countries, an individual’s Government IDs are linked to one another. The stated purpose is for the sake of uniformity and efficiency, and to simplify access to basic rights, services, opportunities, and protection (World Bank, 2019). Many of these Government Identification Systems require biometric data collection. However, some experts have pointed out various ethical and privacy issues that have resulted from the increasing use of biometric identification (Alterman, 2003). For example, the Aadhaar is a 12-digit unique identification number that is issued to all Indian citizens with the aim of standardising data collection and simplify access of services and government subsidies to the enormous population of India (Jain, 2019). To obtain the ‘Aadhaar Card,’ biometric data which include fingerprints, retinal scans, and facial scans, are collected from the citizen (Varun, 2018). However, multiple leaks of sensitive citizen data have occurred over the years, including the sale of personal information on the black market (Tech2, 2018).

According to Clarke (2019), we now live in a world of “digital surveillance economy”, where a massive volume of personal information is acquired and exploited, not just by government agencies for security purposes but also by corporations with the aim of manipulating consumer behaviour, through targeted advertising, to maximise revenue. Ironically, the voluntary and all-pervading espousing of social media and technology has enabled states and corporations to carry out digital surveillance and also misuse people’s personal online information (Westerlund et al., 2021; Taylor, 2002; Odoemelam, 2015; Sekalala et al., 2020). Globally, the justification for state surveillance is based on the argument that society is safer because surveillance prevents illegal and dangerous activities (Clark, 2016; Zhang et al., 2017; Westerlund et al., 2021). However, governments all over the world are facing increased criticism that there is little evidence to support the effectiveness of mass surveillance as a tool to improve security and therefore, does not justify the enormous amounts of personal data being collected through digital surveillance (Westerlund et al., 2021; Zhang et al., 2017; Cayford & Pieters, 2018).

Multiple studies have shown that this loss of control over one’s personal information and the resultant privacy concerns can be significant stressors (Stevic et al., 2022; Dhir & Midha, 2014; Nimrod, 2017; Suh & Lee, 2017). The awareness that they are being watched, tracked, and perhaps profiled, causes people to modify their behaviour and self-censor (Penney, 2016; Boghosian, 2021). Research indicates that self-concealment of one’s thoughts, behaviours and feelings can lead to multiple negative psychological effects like anxiety, depression, and paranoia (Uysal et al., 2010).

Based on previous research, and incidents worldwide, it is evident that privacy, especially when understood as control over one’s personal information, is an extremely important subject that needs further research and analysis. Looking back at the seminal work of Westin (1967), and Article 12 of the Universal Declaration of Human Rights (UN, 1948), it is abundantly clear that the concept of privacy is still central to the development, integrity, and health of both the individual and society (Warren & Brandeis, 1890; Fried, 1970; Rachels, 1975). Moreover, the right to privacy in the digital age has been of specific importance ever since the resolution adopted by the United Nations in 2013 (UN, 2013).

While there is no clear verdict on what the rules and guidelines on privacy should be, the need to study the impact of data privacy on the individual is far from over. The rationale for the current study is based on the theoretical evidence that highlights a significant link between the human right to privacy and its multifaceted impact on individuals, while they try to navigate their everyday lives with increasingly pervasive technology. Therefore, the aim of this research was to study the impact of data privacy on the mental well-being of people and how they perceived the risks and negotiations with their own privacy. To achieve this, the study was conducted on people from different groups and professional backgrounds and thereby identify if some common themes were generated. Using semi-structured interviews and the qualitative method of Thematic Analysis, the study sought to answer the question:

*When considered a Human Right, what is the perceived impact of Data and Information Privacy on Mental Health?*

## Method

### Research approach

The current study was conducted using a qualitative research method, which is useful when trying to understand the meanings and perspectives that individuals attach to their experiences in the world (Hammarberg et al., 2016). The aim was to understand how individuals perceive the impact of Data and Information Privacy on their Mental Health and sense of well-being while they operate in a world where technology and information sharing online is a part of everyday life. Thematic Analysis was used to conduct this research as it is one of the most widely used methods for analysing descriptive data like interviews and can be applied across a range of research questions and epistemological positions (Nowell et al., 2017). It enables the researcher to identify, organise, analyse, and describe the meanings of ‘themes’ (or patterns) that are generated in a descriptive data set (Braun & Clarke, 2006). The combined data from nine interviews was analysed to identify common themes across the responses of all participants.

### Participants

Nine participants were recruited from various professional backgrounds, from an opportunity sample of a larger professional network. The age range of the nine participants was between 27 and 54 and was a mixed sample of male and female participants. The participants belonged to varied social sciences backgrounds such as law, journalism, media, leadership development, photography etc. There were only two requirements to qualify as a participant in this study. The first was to be between the ages of 18 and 60. The second criteria required participants to be educated professionals who use digital technology, services, and social media, thereby enabling them to share their experiences and relationship with Information Sharing and Data Privacy.

### Materials

An interview schedule (Appendix A) was used to conduct semi-structured one-on-one interviews with questions that were open ended and semi structured to allow for more indepth responses and probing. All questions were formulated around participants’ experiences and views on using digital technology and platforms, digital privacy, pros and cons of information sharing online, opinions on biometric data collection, surveillance etc. Interviews were scheduled and conducted only after receiving signed consent forms from each of the participants. Each participant was also sent a debrief sheet after the interview. All interviews were conducted virtually on either Skype or Teams.

### Procedure

Participants were recruited by sharing a detailed advert and poster across professional and social networks. All nine interviews were conducted virtually on either Skype or Teams. No personal details of participants were associated with the data collected. Interviews were recorded and transcribed using the in-built transcription tool of the platform where the interview was conducted. Participants were mailed a debrief sheet upon completion of the interview. Each transcription was compared to the audio recording several times to ensure there were no errors or discontinuity in transcription.

The interview schedule was designed as open ended and semi structured to facilitate rich data gathering from participants, allowing them to share their insights and views in as much detail as they wanted.

Before conducting this study, a detailed ethical application was submitted and approved by the Faculty of Health and Life Sciences Ethics Committee at Northumbria University. This included the design of the study, participant eligibility criteria, recruitment process, research questions and aims, consent forms, information sheet, debrief form, advert used for recruiting and the complete interview schedule.

### Analytic procedure

The six steps of Thematic Analysis (Braun & Clarke, 2006) were applied to analyse the data and write the results. First, the interviews were recorded and transcribed, using the inbuilt transcribing tool of the recording platform. To ensure that there were no errors or discontinuity, the audio recordings were compared several times to the transcription. The researcher familiarised themself with the data by reading all nine transcripts multiple times. For the coding process, sections and phrases from the data were chosen that were relevant to the research question and aims. This resulted in labels or codes describing the highlighted sections. Different colours and symbols corresponded to different codes. Each of these codes described a feeling or idea conveyed by the participants. The third stage entailed identifying patterns in groups of codes wherein each of these groups turned into a theme. Next, the data was revisited to review the generated themes and ensure they reflected the data correctly. Following this, the final themes were given clear and understandable names and then defined. The final step culminated in the writing of this formal report with all the required sections.

## Results and Discussion

**Figure 1:**
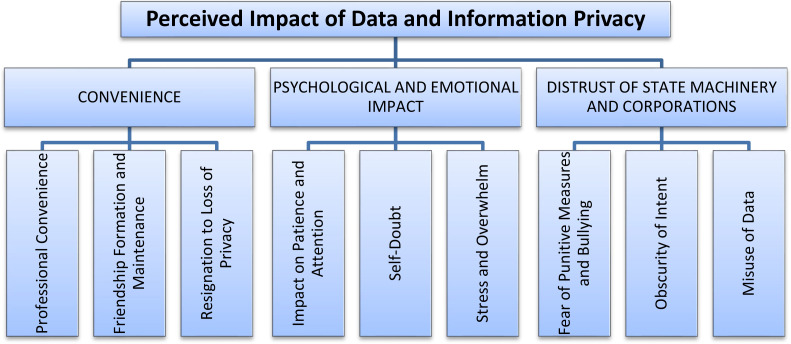
Themes and sub-themes displaying perceived impact of Data and Information Privacy.

The six steps of Thematic Analysis (Braun & Clarke, 2006) were thoroughly followed and applied on the combined data collected from nine different participants. Consequently, three main themes, each containing subthemes, were generated (see Figure 1). The three main themes are:

- Convenience
- Psychological and Emotional Impact
- Distrust of State Machinery and Corporations.

### Theme 1: Convenience

Despite their privacy concerns, each of the nine participants highlighted convenience as one of the primary motivators behind their usage of digital platforms, services, and social networking sites.

#### 1a. Professional convenience

During the interview, participants were asked if it was possible to limit or do without some of these digital services. While the preferred digital service varied, each of the participants described how these were essential for efficiency in their professional work. This included aspects of being able to communicate and share information with colleagues and professional contacts and minimise the logistical costs involved for on-site work places.

*“… absolutely essential…That's the only way we share information with each other within the team…Or we would have to go back to the old traditional model of being on site. The entire team being on site. Collaborating. Much more physically than digitally… But it would lead to many more inefficiencies, it would increase costs significantly…”* (Participant 1, Head of Digital Publication).

*“… as a journalist, it's very hard to go off messaging apps, even if you may want to … you have to cater to the convenience of those who are willing to speak to you… they are not going to go out of their way, well, past a certain degree for you…they'll call you on WhatsApp and you're like, no, no, not on WhatsApp. Then maybe they just won't call you again…”* (Participant 7, Journalist).

#### 1b. Friendship formation and maintenance

Participants talked about how social media sites and other business or common interest networking sites allowed them to meet like-minded people and forge new and lasting relationships. They mentioned how some of these connections developed into real long-lasting friendships. Some of the participants also highlighted that without these platforms, they would have never had any context or opportunity to meet these people because of the different backgrounds and geographical locations. Additionally, while communication applications like WhatsApp were distracting and came with significant privacy concerns, it allowed them to stay in touch with their loved ones who lived in different parts of the world. Participants specified that it would be almost impossible to maintain long distance friendships and relationships without these applications and services.

*“…I have made lots of incredible friends on social media, really people I respect some I work with, so I think positively, a lot of those relationships have, which started as business, have become great buddies… I probably wouldn't have met them otherwise because there would have been no opportunity and no single platform where they would have come and, you know, interacted with me because our worlds are very different…”* (Participant 6, Leadership Development).

*“…for someone who has a lot of friends and people who live very far away, like, like they live in places where it would not be possible for me to visit them…uhh, it does make it easier to not feel that distance as much…”* (Participant 2, Video Journalist)

#### 1c. Resignation to loss of privacy

While most participants agreed that privacy was extremely important and a right that everyone should have, they were acutely aware of how it was becoming increasingly difficult to have that privacy because of data and information sharing online. Understood within the context of the convenience offered by technology and digital platforms, most participants seemed to be reconciled to the fact that the price for this convenience was a loss of one’s privacy.

*“… Personally, I mean as much as it is a right I'd love to have, I think maybe just by virtue of how things are in my profession or whatever. I think I don't have any expectation. I would like it but I don't have an expectation of it anymore… I have accepted the fact that obviously it is no longer a given… I don't think that there is actually any privacy, like everything we are doing online is constantly being tracked…”* (Participant 2, Video Journalist).

While participants were aware of the risks that came with disclosing their information online, it was a risk they were taking in a resigned way, as there seemed to be no way to continue one’s professional work or keep in touch with others and access services, without risking their privacy. However, in the event of an extremely sensitive or confidential conversation, they would choose to have it in person and not digitally. This clearly indicates that participants are quite certain that their privacy is not secure on these applications and that they choose to take these risks and make exceptions on a case-by-case basis.

*“…if with the kind of messaging that I have to do, if it is that super sensitive, then it doesn't even happen uh, on these kind of platforms, uh, it will happen you know, in a one-to-one meeting …”* (Participant 1, Head of Digital Publication).

These views and responses by participants reflect findings of previous research highlighting the conflicted relationship between privacy concerns and the perceived benefits of online information sharing. This is especially important while trying to understand participants’ ‘Resignation to Loss of Privacy.’ Deinlin (2014), defines privacy concerns as a perceived loss of control over our personal information. Several studies show that many online users remain willing to disclose information because they can access information and knowledge they consider relevant and important and because it helps them with resolutions to problems quickly (Hsu et al., 2022; Magedanz & Simões, 2009; Dienlin & Trepte, 2015). People may choose to disclose personal information online when it enables them to maintain relationships and collaborate with others to meet common goals (Hsu et al., 2022.) People also engage in behaviours of such information sharing as it contributes to knowledge and information among other community members (Park et al., 2014).

Communications Privacy is the freedom or right where digitally communicated information should be available and accessible only to the original recipients it was intended for (Rotenberg, 1993; Hung & Wong, 2009). According to Rachels (1975), privacy is essential for us to maintain varied and diverse interpersonal social relationships. Furthermore, intimacy and privacy are deeply related, and privacy is essential for establishing and maintaining intimacy (Roessler & DeCew, 2023; Cohen, 2002; Gerstein, 1978; Fried, 1970; Gerety, 1977). This is consistent with Alan Westin’s concept of intimacy being one of the states of privacy wherein information is only shared with specific people (Westin, 1967). This is demonstrated in the responses of the participants when they mention that they would choose to exchange information in person if the information is confidential and sensitive in nature. Participants’ paradoxical relationship between perceived benefits of online information sharing and privacy concerns is consistent with core theories of privacy. It also highlights the perceived impact of these privacy related decisions on their lives and sense of well-being.

### Theme 2: Psychological and emotional impact

Participants described the complex psychological and emotional effects of information exchange online, while negotiating between the costs and benefits of using digital services.

#### 2a. Impact on patience and attention

Participants described feeling overwhelmed by the sheer volume of information available on the internet. They also talked about how having quick access to so much information, varied viewpoints and content has caused a significant drop in their attention span. Some of the participants highlighted how regularly consuming audio-visual content had made it increasingly difficult to read a book as traditional forms of reading require patience and a longer attention span. They often felt distracted and restless while trying to read or focus on tasks for longer durations. Sometimes, this feeling of distraction and impatience also impacted their relationships with those around them as they were unable to stay present in a moment while communicating with people in person.

*“… the negative impact is obvious. It's all these things are a distraction. They take you away from Life. Take you away from family. Also from work. Because there is something constantly vying for your attention*…” (Participant 1, Head of Digital Publication)

*“… attention span has gone for a toss and that reflects everywhere. It's it's almost like a generational you know, epidemic… so from basic stats on social media, which from what I know there's I think an 8 second attention span now for like I think Instagram and Meta… It's not just social media, attention for reading, attention for people, attention for watching film, attention for, you know, standing in a place… you just want it to be quick, quick, quick…”* (Participant 8, Media Political Consultant).

Most of the participants described the paradox of communication becoming easier and more difficult at the same time, because of communication applications like WhatsApp, email etc. For example, as discussed for the theme of Convenience, texting and communication platforms allow us to stay in touch with people far away and establish an instant communicative connection. However, at the same time, because of their virtual nature, they also lead to miscommunication and impatience due to the absence of non-verbal cues. Furthermore, the knowledge that communication and responses can be made instantly available due to technology, leads to increased impatience and distress if people do not receive a response immediately.

*“… But I think instant communication also at times is a challenge in itself…the ease with which you can connect with, if that's a problem, the other side of the problem is also the ease with which you can disconnect… If the person instantly disconnects for whatever reason, it does give you a sense of a jolt for a second… Oh, what happened?…”* (Participant 5, Photographer and Filmmaker)

Several studies and research show that information overload or too much information is an actual problem which can cause more hindrance than help and can have multiple negative effects on individuals (Bawden & Robinson, 2020). This overload can also cause symptoms like a loss of attention span (Hallowell, 2005), cognitive overload (Kirsch, 2000) and a reduction in productivity manifesting as an inability to focus or concentrate (Rose, 2010). The participants of the current study display all these symptoms including symptoms as specific as a growing inability to read longform reading material (Carr, 2010). Research by Walsh (2012), highlights how being constantly connected to communication and information via devices causes a psychological state of ‘always on’ and creates this perceived sense of overload and ‘information imposition’ which, perhaps, the individual did not consciously seek.

#### 2b. Self-doubt

Most of the participants in this study use digital platforms to further their professional goals and agreed that it allowed them to showcase their work and engage with others for exchange of knowledge and ideas. While participants agreed regarding the professional and intellectual benefits, it came with other negative effects like feelings of inadequacy about their own authenticity and competence. Despite their objective success, they felt that they often compared themselves to others because the same platforms expose them to other people’s accomplishments. This comparison was not just limited to one’s immediate peers, as social media allows us to compare ourselves to millions of people from all over the world by exposing us to constant content.

*“… this insecure constant comparison sometimes tends to shift focus from what you are doing to what others are doing… and so I think that is one of the things which I feel, the constant state of comparison makes you feel that somewhere you're losing out…”* (Participant 5, Photographer and Filmmaker).

*“…there's always a sense of comparison, because you see all these people looking a certain way, always looking Perfect. But, uh, you know, very manicured, kind of lifestyle and everything. And it feels like somehow you, you are inadequate in that space…”* (Participant 2, Video Journalist).

Several participants talked about how their exchange of ideas and knowledge on social media made them reflect on their own motivation behind it. They described how the process of introspection made them realise that a lot of times, this exchange of ideas was perhaps just for validation in a quest to appear intelligent and win arguments. Prior to the honest introspection, these exchanges were causing participants stress and anxiety as they were caught up in the performative actions that led to feelings of validation.

These feelings and experiences of the participants are consistent with previous research on the effects of information and interaction on social networking sites. Whenever people are confronted with information about others and their achievements, they tend to relate it to themselves (Mussweiler et al., 2005). Digital platforms expose people to enormous amounts of information about others and provide multiple avenues and sources of communication and information and thereby greatly increase the frequency of comparison to others (Lee, 2014). Research by Goulding (2001) indicates that this overload of information causes symptoms like fatigue, anxiety, diminished analytical capacity and self-doubt.

#### 2c. Stress and overwhelm

Most of the participants described feeling overwhelmed and anxious because of the enormous volume of information, ideas, and news that they must deal with daily. They mentioned how the spectrum of information went from light entertainment videos all the way to deeply disturbing videos on conflicts and war in different parts of world, all within a very short span of scrolling on the internet. This causes exhaustion and fatigue but also a plateauing of emotions as there is no time to process the range of emotions that a person should ideally experience when encountering such vastly different types of information.

*“… you see what's happening in Gaza and you see that people are dying…You see that now those are people who need your help….. You see that and within the same second you are seeing like someone cooking spaghetti and saying, oh, you know, like I have the best diet for you… You don't have a second to process anything, right?… Like there is a complete plateau in emotion…”* (Participant 8, Media Political Consultant)

As most participants are dependent on these platforms for their professional work, verifying the authenticity of content, news and information is imperative. Given their awareness of the internet being plagued by fake news, misinformation and deep fakes, participants mentioned how having to verify every bit of information also added to their levels of stress and fatigue. Additionally, the need to always check privacy settings to avoid misuse of one’s personal information, also added time, stress and anxiety.

*“……. Like we can’t know or believe if a video is true or not. You don't know. We don't know if somebody… you’re just talking and they can patch it up and you bring in some software. You can't trust anything you know, actually. Yeah. So it's also increasing trust issues. You don't know what's real or fake anymore…”* (Participant 4, Legal Professional)

Existing research highlights how misinformation and fake news further compound the problem of information overload and fatigue as there are far too many sources of information and news on the internet (Bawden & Robinson, 2020). Having to determine their validity and authenticity in order to avoid fake news and misuse of one’s personal information adds more stress and anxiety to an already stressful situation where there is too much information to handle and process (Bawden & Robinson, 2020; Wurman, 2001; Anderson & Raine, 2017; Kovach & Rosenstiel, 2011; Schmitt et al., 2018). Additionally, such vast amounts of highly diverse and varied information which need to be processed and understood lead to a sense of lowered well-being and feelings of depression and unhappiness (Kominiarczuk & Ledinska, 2014).

Participant responses highlight the importance of the functions of privacy, such as self-evaluation and personal autonomy, as described by Westin (1967). These functions allow an individual to protect oneself from being exposed to or manipulated by others and also enable self-reflection and processing of one’s experiences. This has become increasingly challenging to achieve due to such intrusive and allpervasive exposure to information and communication (Marky, 2023). Too much information and contact can feel like an invasion of the self and consequently impact one’s sense of well-being (Pedersen, 1997). The paradox of how much information and communication to expose oneself to, is often a complicated process requiring self-reflection and introspection. Evidently, the complex nature of privacy as a human right has varied effects on the mental well-being of an individual.

### Theme 3: Distrust of state machinery and corporations

When discussing their doubts and inhibitions surrounding their privacy and online personal information, all participants expressed a clear distrust in the authorities and agencies responsible for collecting and handling their data. This was a recurrent thought that was present across all the nine interviews.

#### 3a. Fear of punitive measures and bullying

Although participants were from different professions, each of them shared experiences of facing bullying and backlash for sharing their political and social opinions on the internet. The bullying and aggression on these platforms usually came from supporters of political organisations. Participants mentioned that this was often the case whenever they engaged in discussion or commentary which questioned or criticised some specific political parties, public policies, or establishments. Furthermore, they witnessed that nothing was being done to control this abusive behaviour on these platforms owned by private corporations. Threats ranged anywhere from hateful comments to threats like rape and physical violence, indicating that the bully could access the physical location and other personal information of the participant. And while the threats did not always translate into real life harm, participants felt that these threats could not be taken lightly and therefore caused fear and distress.

*“… in a situation where you know that many of these people abusing you do have the backing of a lot of power. Uhh often government power. Sometimes other kinds of power, and so the threat doesn't necessarily always feel like it's minute or inconsequential like sure, if someone you know says something like I'm going to come to your house rape you, you don't necessarily believe that they're going to show up at your house and rape you. But you do know that their threat is being seen as valid by a large section like, ‘Yeah, that's what you deserve’…”* (Participant 7, Journalist)

Some of the participants, especially journalists, mentioned facing punitive action for questioning or criticising certain organisations and public policies. They felt this was made possible because of their personal information, privacy and confidentiality being compromised online. They also emphasised how this made it especially difficult for them to work in their profession as privacy is an essential aspect of being able to work as a journalist.

*“… if you've taken my laptop away you have all my information that compromises my work, uh like it compromises, not just my privacy, but compromise my work and my sources, because then it makes me unreliable and then in the future it becomes harder for people to trust me. For no fault of mine. Because they will feel that if my privacy is compromised and therefore by working with me, their privacy will be compromised…”* (Participant 2, Video Journalist).

The experiences and views shared by the participants clearly indicate how digital platforms and self-exposure online enable bullying, harassment, and intimidation. Multiple studies indicate that cyber bullying and sexual harassment is a global problem where information about an individual is used to harass and intimidate them online (Mesch, 2009; Whittaker & Kowalski, 2014; Kowalski et al., 2012). Furthermore, threats and harassment directed at journalists online for their work and reporting is becoming far more prevalent in the digital age where bullies can access personal information online about their victims (CNTI, 2024; Lewis et al., 2020; Waisbord, 2020). Digital platforms allow perpetrators to remain anonymous and attack women and other marginalised groups through provocative messages and threats (Ferrier &Garud-Patkar, 2018).

Existing research and investigation also show that journalists routinely face threats of physical violence, imprisonment, and intimidation to an extent where they may be compelled to leave their professions altogether (Binns, 2017; Posetti et al., 2021; CNTI, 2024).

#### 3b. Obscurity of intent

Participants were asked if they felt safer because of CCTV cameras (closed-circuit television), surveillance and facial recognition in public spaces. While some mentioned that they understood the benefits being specific to deterrence of crime, most participants did not necessarily feel convinced about the stated purpose of cameras and facial recognition in public spaces and also felt uncomfortable with being watched. This discomfort came from the fact that there was no way to know who had access to all the footage and where it was being stored. They also described feeling very uneasy about surveillance, knowing that it could be misused by anyone who has access to it, including for the purpose of racial, gender, and many other types of social profiling.

*“…But who are you watching? And what are you watching? Well, why are you watching? Where is this being stored? Who has it? You don't know. My problem is that you don't know. I feel like people are constantly kept in the dark…”* (Participant 8, Media Political Consultant)

*“…it provides a bigger, a foundational base for mass surveillance…where people are very different, trying to exist and flourish together, but at the same point in time have very deep-seated caste, gender, social geographic biases against each other or for each other…”* (Participant 9, Lawyer)

Some of the participants also mentioned how it felt like an intrusion of their privacy. Additionally, the knowledge that most of their personal information and biometrics was ‘out there’ on digital platforms, public facial recognition and camera surveillance compounded this feeling of intrusion. It made them feel very conscious and aware of how they behaved all the time in public and inhibited their natural behaviour.

*“… like it makes feel you restricted because you feel like you're constantly being watched. Not in a dramatic movie sense. But like, everything you do, someone's watching it, someone's got information on you, on there. And then when your face is bare and like your other information is all over the place, it just, Yeah, it just doesn't feel as safe as they make it seem like it is…”* (Participant 3, Media Manager).

When asked about their views on collection of biometric data, participants said they understood that the stated objective is to simplify access to various services by centralising each individual’s personal information. However, participants felt hesitant and anxious about the mass collection of biometric data. They mentioned that their doubts and distrust came from the fact that there was very little information on how and where this biometric data was being stored and also because there were vast inefficiencies and discrepancies on ground about the stated purpose being achieved.

*“…the government has much more power and can cause much more damage to individuals, groups, especially those who are marginalized and may not know how to, you know avail any kind of remedy or seek accountability. So I feel deeply uncomfortable about it. Biometrics also, you know they don't work in the way they are stated to work and it provides a bigger, a foundational base for mass surveillance…”* (Participant 9, Lawyer)

The unease, ambiguity and doubt participants reported are consistent with existing research on surveillance and data collection. As pointed out by Clarke (2019), we live in an age of ‘digital surveillance economy’ where a massive amount of citizens’ personal information is collected and exploited by government agencies and corporations alike for security purposes and profit maximisation. While globally the justification for surveillance is the safety, security and autonomy of citizens, governments all over the world are facing intense criticism for providing little to no evidence in proving the effectiveness of this mass surveillance in achieving these stated purposes (Clark, 2016; Zhang et al., 2017). Furthermore, the knowledge that one is being tracked, watched and perhaps also profiled causes people to self-censor and modify behaviour (Penney, 2016; Boghosian, 2021) which consequently leads to other negative behaviours and feelings such as anxiety, depression and also the phenomenon known as ‘the chilling effect’ (Solove, 2007; Kaminski & Witnov, 2014; Uysal et al., 2010). In another recent study, it was found that government surveillance can have a negative impact on democratic processes like freedom of speech and the right to protest due to fears of profiling and punitive measures (Murray et al., 2024).

#### 3c. Misuse of data

Participants did not trust private corporations to have the safety or privacy of individuals at heart. All the participants were of the firm opinion that corporations operated on a profit model and therefore, the personal and privacy interests of individuals was not of much value to these private companies. This is also relevant to the theme fear of punitive measures and bullying discussed above where participants mentioned how action was rarely taken to stop abusive behaviour on these sites or when abuse was made possible because data privacy had been compromised. Participants mentioned how their personal information and online data was most likely being compromised or sold to third parties and therefore, identity theft, financial fraud, scams, and false advertising were major concerns to be wary of.

*“…Because these are corporations. So they work with a profit motive. If having a fraudulent advertiser on their platform makes them money, then they don't care about the fact that you lose money. It’s as simple as that…”* (Participant 1, Head of Digital Publication)

This fear and distrust extended to the biometrics collected by government agencies because of multiple leaks and data breaches having occurred globally. Participants shared how there was no way to know or control how this data was being stored and shared forward and therefore, offered very little recourse in the event of a misuse of data.

*“… I don't think there's much recourse for the citizen in this, because if something like a leak happens, your data gets stolen and it's misused. Well, I mean, in the best-case scenario, the person you can hold accountable is the person who stole it, but you still can't hold accountable the company that had it and stored it in a, you know, unsafe way to, God knows what end…”* (Participant 7, Journalist).

Some participants also explained how all online activity, spending patterns, location history, browsing history and personal information is potentially used to know everything about the individual and can be easily used to profile people and manipulate behaviours. This manipulation could range anywhere from targeted advertising for products, all the way to influencing what type of political and ideological content one is exposed to.

*“…I'm getting profiled in those categories that this maybe you know this person who is a female in this age category and we also understand that these are her interests by my other digital footprints, is of this kind of opinion… you categorise them into groups and you see OK, this is the pattern. You find patterns. And then you try and strategize how to manipulate these patterns into thinking in a certain way…”* (Participant 4, Legal Professional)

These responses indicate a strong distrust and wariness that participants felt about private corporations and state agencies not safeguarding people’s privacy and most likely also misusing personal information and data from online activity, without any explicit consent to do so.

Research and findings from events worldwide indicate that these fears and inhibitions expressed are valid and not without reason. Several experts have pointed out the various ethical and privacy concerns associated with the use of biometric identification (Alterman, 2003). As discussed earlier in the introductory section of this study, globally there have been multiple incidents, including the United States and India, where massive data leaks of citizens’ sensitive information have occurred (Marcus, 2018; Marinos & Clements, 2018; Tech2, 2018). The voluntary and all-pervasive use of technology has allowed government agencies and private corporations to carry out widespread mass surveillance and manipulate people by misusing their personal information online (Westerlund et al., 2021; Taylor, 2002; Odoemelam, 2015; Sekalala et al., 2020). As was revealed by the Cambridge Analytica Facebook scandal of 2018, misuse of personal information on platforms owned by private corporations could go as far as harvesting user information for psychographic profiling and targeted political advertising for a national election (Cadwalladr, 2018; Lapowsky, 2019).

As described in Alan Westin’s seminal work on privacy, one of the functions of privacy is limited and protected communication which is described as setting interpersonal boundaries and protecting the exchange of information with those we trust (Westin, 1967). The findings of the study are consistent with existing research on the importance and complexities of privacy, especially when considered a human right. In trying to answer the research question of this study, the lived experiences and opinions of the participants provide deep insight into how they feel impacted by Data and Information Privacy.

## Conclusion

### Summary of Findings

1. All participants reiterated that digital platforms and services were essential for professional efficiency, cost reduction, information exchange and collaboration with colleagues. Additionally, these platforms facilitated and enabled formation and maintenance of friendships/relationships with like-minded people and loved ones. However, participants were acutely aware of how risky these services were for their privacy. The loss of privacy and personal information was a price that participants seem to be resigned to while weighing the benefits of these services and they made exceptions on a case-by-case basis.
2. Participants described feeling overwhelmed by high volumes of information online. Constant access to varied viewpoints and frequent consumption of audio-visual content resulted in loss of attention span, patience and a struggle while reading books and long form material. The same impatience and inability to stay present in a moment for long, manifested while communicating with people in person.
3. Participants reported feelings of self-doubt and inadequacy caused by having access to information about other people’s accomplishments. While digital services allowed them to collaborate and acquire knowledge, it also inflicted comparisons from people all over the world. These effects were mitigated only after intentional introspection and self-reflection.
4. Enormous volumes of information and news also caused anxiety, fatigue and a plateauing of emotions as the quick moving and contrasting types of news and information doesn’t allow for processing of so many emotions occurring all at once. Additionally, the presence of fake news, deep fakes and data leaks increases levels of stress and overwhelm in participants as they have to constantly worry about verifying all information for authenticity, while also trying to safeguard their personal information.
5. All participants reported facing varying degrees of bullying and aggressive intimidation on digital platforms while sharing their social or political views and during their professional reportage. They all reiterated that there was never any control of such bullying on privately owned digital platforms. Even though threats did not always translate to real life harm, participants believed that they couldn’t be taken lightly and consequently this caused a looming sense of fear and distress. Misuse of data by corporations enabled intimidation and manipulation in varied situations. Additionally, participants did not trust corporations to store and use personal information ethically nor value the safety and privacy of the individual. Similarly, participants also had serious concerns about how the stated intentions of biometrics collection and mass surveillance did not always match the results on ground.

### Strengths and Limitations

The current study aimed to gain insight into how individuals experienced and felt about sharing their information online within the context of privacy being an important human right. The semi-structured one-on-one design of the interviews allowed participants to share their experiences, feelings, and opinions in detail. It also enabled probing whenever participants wished to offer further insight beyond what was asked in a question (Robinson, 2023). The findings of this study were reflective of research from across the world, notwithstanding the specific geographical locations of the participants. This is very evident from the supporting literature provided in the results section of this study. Research indicates that globally, many people remain unaware of the privacy risks and concerns resulting from engagement with digital services (Nguyen et al., 2023). However, the participants of this study were highly educated and well informed of the risks, dangers and ethical concerns surrounding digital privacy. This high level of awareness allowed them to share deep insights into the complexities of the privacy paradox, which is defined as the engagement in careless behaviour online despite being aware of the risks involved (Barth et al., 2019). The participants of this study were able to share in-depth insights and personal experiences which described certain negative effects on their life like stress, self-doubt, fear, and anxiety. Additionally, their professional knowledge on subjects like ethical violations, manipulation, and mismanagement of data by government agencies and private corporations, further enabled them to understand their own fears and inhibitions clearly. Participants had well defined views on the importance of privacy for individuals and society as a whole. As described in Westin’s theories on privacy (Westin, 1967), participants navigated their privacy concerns in a self-reflective way where they regularly examined how their mental and emotional well-being was being impacted due to disequilibrium in their state of privacy. They were able to introspect and analyse the importance and impact of aspects of privacy such as anonymity, intimacy, and personal autonomy (Marky, 2023). However, if the study is replicated on a different sample, the results may be quite different if the participants are unaware of the risks and ethics of privacy and/or lacking in self-reflective skills. Furthermore, while most participants in the current study were expressive and descriptive in their responses, there was some degree of hesitation and self-censorship in naming specific organisations or exact details of the forces that contributed to the negative effects and experiences. Ironically, privacy concerns and fear of punitive measures is perhaps one of the main reasons for this while self-censorship itself is one of the negative effects of privacy concerns.

### Conclusions

The findings of this study highlight the growing sense of deep distrust and the privacy and ethical concerns associated with social networking sites, digital platforms, along with the dubious relationship these platforms have with various agencies (Taylor, 2002; Odoemelam, 2015; Sekalala et al., 2020; Westerlund et al., 2021).

There are many critiques of the importance of privacy and much disagreement on it being an absolute right. Some experts argue that the right to privacy is merely a cluster of rights and not absolute (Thompson, 1975). Furthermore, there are vast differences in systems of privacy protection in the Unites States and Asia as compared to the European Union. (Newman, 2008). A lack of consensus notwithstanding, privacy concerns and the ethics associated with them have massive implications worldwide (Alterman, 2003; Clark, 2016). Access to digital services and online connectivity is almost imperative to citizens’ growth and development in the present era but at the same time there need to be clear safeguards that protects an individual’s privacy while accessing the same technology (UNHRC, 2022; UN, 2013). This complicated interplay of access to knowledge vis a vis protection of privacy is one of the major challenges that has been discussed and highlighted in the 2020 United Nations’ Secretary General’s report (UNSG, 2020). It imperative that research and analyses of these conflicting challenges must continue.

The findings of this study further reiterate the role privacy plays in impacting the well-being of individuals and society. Privacy is fundamental to human growth and development (Fried, 1970). Furthermore, privacy is also a necessary condition for democratic processes such as freedom of speech, right to protest and also the right to safe access to services (Richards, 2013; UN, 1948; UNSG, 2020; UN, 2013). It is very evidently a right and concept that needs continued research as it is central to the development and well-being of humans and the society they inhabit.

## Action editor

Lars-Gunnar Lundh served as action editor for this article.

## Open access

This article is distributed under the terms of the Creative Commons Attribution 4.0 International License (http://creativecommons.org/licenses/by/4.0/), which permits unrestricted use, distribution, and reproduction in any medium, provided you give appropriate credit to the original author(s) and the source, provide a link to the Creative Commons license, and indicate if changes were made.
